# Notwithstanding Circumstantial Alibis, Cytotoxic T Cells Can Be Major Killers of HIV-1-Infected Cells

**DOI:** 10.1128/JVI.00306-16

**Published:** 2016-07-27

**Authors:** Saikrishna Gadhamsetty, Tim Coorens, Rob J. de Boer

**Affiliations:** a Theoretical Biology, Utrecht University, Utrecht, The Netherlands; b University College Utrecht, Campusplein, Utrecht, The Netherlands; Emory University School of Medicine

## Abstract

Several experiments suggest that in the chronic phase of human immunodeficiency virus type 1 (HIV-1) infection, CD8^+^ cytotoxic T lymphocytes (CTL) contribute very little to the death of productively infected cells. First, the expected life span of productively infected cells is fairly long, i.e., about 1 day. Second, this life span is hardly affected by the depletion of CD8^+^ T cells. Third, the rate at which mutants escaping a CTL response take over the viral population tends to be slow. Our main result is that all these observations are perfectly compatible with killing rates that are much faster than one per day once we invoke the fact that infected cells proceed through an eclipse phase of about 1 day before they start producing virus. Assuming that the major protective effect of CTL is cytolytic, we demonstrate that mathematical models with an eclipse phase account for the data when the killing is fast and when it varies over the life cycle of infected cells. Considering the steady state corresponding to the chronic phase of the infection, we find that the rate of immune escape and the rate at which the viral load increases following CD8^+^ T cell depletion should reflect the viral replication rate, ρ. A meta-analysis of previous data shows that viral replication rates during chronic infection vary between 0.5 ≤ ρ ≤ 1 day^−1^. Balancing such fast viral replication requires killing rates that are several times larger than ρ, implying that most productively infected cells would die by cytolytic effects.

**IMPORTANCE** Most current data suggest that cytotoxic T cells (CTL) mediate their control of human immunodeficiency virus type 1 (HIV-1) infection by nonlytic mechanisms; i.e., the data suggest that CTL hardly kill. This interpretation of these data has been based upon the general mathematical model for HIV infection. Because this model ignores the eclipse phase between the infection of a target cell and the start of viral production by that cell, we reanalyze the same data sets with novel models that do account for the eclipse phase. We find that the data are perfectly consistent with lytic control by CTL and predict that most productively infected cells are killed by CTL. Because the killing rate should balance the viral replication rate, we estimate both parameters from a large set of published experiments in which CD8^+^ T cells were depleted in simian immunodeficiency virus (SIV)-infected monkeys. This confirms that the killing rate can be much faster than is currently appreciated.

## INTRODUCTION

The role that cytotoxic T cells (CTL) play in controlling human immunodeficiency virus type 1 (HIV-1) infection is poorly understood (1, 2). Genetic associations with a limited number of protective human leukocyte antigen (HLA) alleles (3) suggest that they can control the infection to very low viral loads in a small subset of patients called “elite controllers.” The fact that, during acute infection, HIV-1 tends to evolve several immune escape mutations suggests that in this early phase, there is a strong selection pressure to evade the CTL responses (4–7; but see Roberts et al. [8]). Finally, the depletion of CTL with monoclonal antibodies to CD8 leads to marked increases in the viral load (9–15). CTL can protect by killing infected cells and/or by various nonlytic mechanisms, including the secretion of gamma interferon (IFN-γ) and macrophage inflammatory protein 1α (MIP-1α) and MIP-1β (16, 17, 18). The relative contributions of these two mechanisms in controlling HIV-1 infection are debated (11, 18–26).

Several lines of evidence suggest that CTL hardly kill CD4^+^ T cells that are productively infected with HIV-1. First, the death rate of productively infected cells was estimated by the initial downslope of the viral load during successful antiretroviral treatment (ART) (27, 28); this downslope, δ, is remarkably independent of the viral load and the CD4^+^ T cell count (29) and is currently estimated to be about δ = 1 day^−1^ (30). If this downslope indeed reflects the rate at which productively infected cells die, the killing rate would have to be slower than one per day (31, 32). Second, and even more striking, it was shown that the prior depletion of CD8^+^ T cells by monoclonal antibodies hardly affects the downslope of the viral load during ART (11, 12). Hence the death rate, δ, of productively infected cells is hardly influenced by the absence of CD8^+^ T cells, which suggests that CTL hardly kill, and that the major effect of CTL is nonlytic (11, 22, 24). Similarly, during acute infection, the downslope following the peak in the viral load is hardly affected by the presence of many cognate CD8^+^ T cells (33, 34), and it is puzzling why the peak viral load, which is a measure of poor immune control, correlates positively with the downslope (35). Third, the very low rate at which most viral mutants escaping a chronic CTL response take over the viral quasispecies (8, 36) suggests that the CTL response kills only a small fraction of the productively infected cells (36). Additionally, cells infected with virus that have escaped a CD8^+^ T cell response do not live longer than cells infected with wild-type virus (20). These data have typically been analyzed with the conventional model of HIV-1 infection, and this modeling confirms that CTL-mediated killing rates have to be much slower than one per day. Since CTL also have nonlytic effects (16–18), these slow killing rates could be true and be consistent with an important role of CTL in the control of HIV-1 infection.

Our main result is that these interpretations need to be revised when the conventional model is extended with an eclipse phase preceding the stage during which infected cells actively produce virus particles. Whenever cells in the eclipse phase and cells actively producing virus differ in their susceptibility to killing by CTL, this extended model requires much faster killing rates. To compensate for the slow killing in the unsusceptible phase, the killing in the other phase has to be relatively fast. In the extended model, neither the viral downslopes, δ, nor the immune escape rates are expected to reflect the killing rate. We show that the rapid upslopes of the viral loads that were observed following the depletion of CD8^+^ T cells (9–12, 14, 15) should reflect the effective replication rate, ρ, of the virus at the chronic set point. Since the killing rate should balance this replication rate during the chronic steady state, we can compute the killing rates from the observed upslopes of the viral loads. The rapid killing rates that we obtain are perfectly consistent with the apparently contradictory lines of evidence discussed above. Because the extended model with an eclipse phase is more realistic than the conventional model and readily explains why the observed viral downslopes are independent of the viral load and the CD4^+^ T cell count (29), our conjecture is that, if CTL protect by killing infected cells, these killing rates are much faster than is currently appreciated.

## MATERIALS AND METHODS

### Conventional one-stage model.

Consider the general model for an HIV-1 infection, with CD4^+^ target cells (*T*), productively infected cells (*I*), virus particles (*V*), and *n* clones of cognate CD8^+^ T cells (*E_i_*), i.e.,

(1) $$\frac{\text{d}T}{dt}=F\left(T\right)-bTV$$

(2) $$\frac{\text{d}I}{\text{d}t}=f\;bTV-d_{I}I-I\overset{n}{\underset{i}{\sum }}k_{i}E_{i}$$

(3) $$\frac{\text{d}V}{\text{d}t}=pI-d_{V}V$$

(4) $$\frac{\text{d}E_{i}}{\text{d}t}=G\left(E_{i},V\right)-d_{E}E_{i}$$

for *i* = 1,2,…,*n*, and where *F*(*T*) and *G*(*E_i_*,*V*) are functions defining the production of target cells and activation of immune effector cells. The parameter *b* is the infection rate, *f* is the fraction of cells that become productively infected, *d_I_* is the normal death rate of productively infected cells, *k_i_* is a mass action killing rate, *p* is the rate at which productively infected cells produce virus, *d_V_* is the rate at which virus is cleared, and *d_E_* is the death rate of cognate CD8^+^ T cells. After making the conventional quasi–steady-state assumption (QSSA), d*V*/d*t* = 0, we study the behavior of this system around its steady states by considering the core viral replication cycle defined by equation 2. By assuming that the dynamics of target cells and immune effector cells are slower than those of the productively infected cells, we replace *T* and $\overset{n}{\underset{i}{\sum }}k_{i}E_{i}$ in equation 2 by the presumed slow “constants” *T̄* and *K*, respectively, and observe that the core of viral replication becomes a linear system obeying

(5) $$\frac{\text{d}I}{\text{d}t}=I\left[f\;\beta \overset{¯}{T}-d_{I}-K\right],\;\text{with}\;\text{the}\;\text{solution}\;I\left(t\right)=I\left(0\right)e^{\lambda t}$$

where λ = *f* β*T̄* − *d_I_* − *K*, β = *bp*/*d_V_*, and *V* = (β/*b*)*I*.

We define the effective replication rate of the virus as ρ = *f* β*T̄ −* d_*I*_ and observe that steady state requires that the killing rate balances replication, i.e., *K* = ρ. The fact that the killing rate should reflect the viral replication rate in this very basic model generalizes several earlier papers arguing that the magnitude of the cellular immune response need not be reflected in the death rate of productively infected cells (19, 37–39). The replication rate of the virus during the initial phase of an acute infection is defined by ρ(0) = *f*β*T*(0) − *d_I_*, and the initial downslope of the viral load during an effective antiretroviral therapy (ART) setting β = 0 is δ = *d_I_* + *K*. Both have been estimated previously. Two recent studies in acutely infected patients estimate that ρ(0) ≃ 1 day^−1^ (40, 41), whereas an early study based on a limited number of patients and time points estimated that ρ(0) ≃ 1.6 day^−1^ (42). The latter resembles the replication rate found in macaques (34, 43–45). Since we will also consider the viral replication rate following the depletion of CD8^+^ T cells, which cannot be done in patients, we will parameterize our model on macaques by considering 1 ≤ ρ(0) ≤ 1.5 day^−1^ and discuss how the results translate to the probably somewhat slower viral replication in humans. The initial downslope of the viral load, δ, has been estimated in humans (27, 28, 46), and more recent estimates using combinations of drugs that better suppress residual viral replication (30) suggest that this downslope varies around δ ≃ 1 day^−1^. Very similar downslopes have been found in monkeys (11, 12, 45). Following the depletion of CD8^+^ T cells, which sets *K* = 0, the viral load will increase at its effective replication rate, ρ, which in this model reflects the killing rate before depletion because ρ = *K* (22). The initial replication rate of a viral mutant escaping 1 out of *n* equal immune responses and experiencing a fitness cost, *c*, in the chronic steady state is

(6) $$\lambda \prime =\rho \left(1-c\right)-\frac{n-1}{n}K=\rho \left(\frac{1}{n}-c\right)$$

The mutant is therefore only expected to take over whenever the fitness cost is smaller than the inverse breadth of the immune response, i.e., *c* < 1/*n*. The rate, λ′, at which immune escape mutants are expected to replace the wild type should therefore decrease when the breadth, *n*, of the immune response increases over the course of an infection (6, 36, 38, 39).

### Two-stage model.

Several of these well-known kinetic properties of the one-stage model change when we allow for an eclipse phase of the infected cells. Let us therefore split the infected cell population of equation 2 into a subpopulation of recently infected cells that are not yet translating viral mRNA or producing virus (*I*_1_) and a subpopulation of productively infected cells (*I*_2_) that are actively producing virus, i.e., d*V*/d*t* = *pI*_2_ − *d_V_V*. By the same QSSA, we now obtain that β = *bp*/*d_V_* and that *V* = (β/*b*)*I*_2_, which leads to the model

(7a,b) $$\frac{\text{d}T}{\text{d}t}=F\left(T\right)-\beta TI_{2},\;\frac{\text{d}E_{i}}{\text{d}t}=G\left(E_{i},V\right)-d_{E}E_{i}$$

(8a,b) $$\frac{\text{d}I_{1}}{\text{d}t}=f\;\beta TI_{2}-\left(d_{1}+\gamma +\overset{n}{\underset{i}{\sum }}k_{1_{i}}E_{i}\right)I_{1},\;\frac{\text{d}I_{2}}{\text{d}t}=\gamma I_{1}-\left(d_{2}+\overset{n}{\underset{i}{\sum }}k_{2_{i}}E_{i}\right)I_{2}$$

In this model, 1/γ defines the average length of the eclipse phase, which is about 1 day (Table 1), and *d*_1_ and *d*_2_ represent the normal death rates of infected cells. The breadth of the immune response is again defined by *n* clones of CTL, and by setting the killing rates, k_1_i and k_2_i, we can define whether an immune response acts early (k_1_i__ > 0) and/or late (k_2_i__ > 0). We again study the behavior of this system around its steady states by considering the core viral replication cycle defined by equation 8. Replacing *T* and the two summation terms in equations 8a and b by the presumed slow “constants” *T̄*, *K*_1_, and *K*_2_, respectively, the core again becomes a linear system, now obeying

(9) $$\left(\begin{array}{c} \text{d}I_{1}/\text{d}t\\ \text{d}I_{2}/\text{d}t \end{array}\right)=\left(\begin{array}{cc} -d_{1}-\gamma -K_{1} & f\;\beta \overset{¯}{T}\\ \gamma & -d_{2}-K_{2} \end{array}\right)\left(\begin{array}{l} I_{1}\\ I_{2} \end{array}\right)$$

Since the core model of equation 8 is linear and has no feedbacks, the stable chronic steady state of the full model is established by the feedbacks in equations 7a and b, approaching a target cell availability, *T̄*, and killing rates, *K*_1_ and *K*_2_, that perfectly balance the effective viral replication rate.

**TABLE 1:** Parameter setting of the two-stage model^*a*^

Parameter	Value (day^−1^, unless otherwise noted)	Description
*d_T_*	0.1	Death rate of CD4^+^ target cells (range, 0.01–1; see Fig. A1)
*s*	0.1	Daily production of target cells (scaled, *T̄* = *s*/*d_T_* = 1)
*d_E_*	0.01	Death rate of CD8^+^ effector (and/or memory) T cells
*p*	1.01	Proliferation of CD8^+^ effector T cells (*p* − *d_E_* = 1 day^−1^ [44])
γ	1	1/γ is the avg length of the eclipse phase [59, 60, 49]
*k_i_*	1–100^*b*^	Maximum killing rate
ρ(0)	1.5	Initial viral replication rate (see equation 12)
*h_i_*	0.001–0.01^*b*^	Half-saturation constant of the immune response
*f*	1^*b*^	Fraction of cells surviving initial infection
*d*_1_	1 or 0.1	Death rate of cells in the eclipse phase (early or late)
*d*_2_	1 or 2	Death rate of productively infected cells (early or late)
β	8.75 or 9.1	Infection rate: equation 13 for ρ(0) = 1.5 day^−1^ (early or late)

a To remain consistent with the observed viral downslopes, δ ≃ 1 day^−1^, during ART, we use equations 14a and b to parametrize the model slightly differently when the killing is largely early or mostly late (see the text). For simplicity, all cells survive the initial infection in the two stage-model (i.e., *f* = 1); we consider abortive infections (52, 53), i.e., *f* = 0.1, in the three-stage model.

b Not per day.

The general solution of equation 9 obeys $\left(I_{1}\left(t\right),I_{2}\left(t\right)\right)=C_{1}v_{1}{\text{e}}^{\lambda_{1}t}+C_{2}v_{2}{\text{e}}^{\lambda_{2}t},$ where λ_1,2_ are the eigenvalues of the matrix in equation 9, **v**_1,2_ are the corresponding eigenvectors, and *C*_1,2_ are integration constants. Since the viral load remains proportional to the density of productively infected cells, *V* = (β/*b*)*I*_2_, this solution also provides the viral load following a perturbation of the steady state as $V\left(t\right)=c_{1}{\text{e}}^{\lambda_{1}t}+c_{2}{\text{e}}^{\lambda_{2}t},$ where the constants *c*_1,2_ are complicated combinations of the parameters and the initial conditions, obeying *c*_1_ + *c*_2_ = *V*(0). The two eigenvalues are defined as

(10) $$\lambda_{1,2}=\frac{1}{2}\left(-\left(d_{1}+\gamma +K_{1}+d_{2}+K_{2}\right)\pm \sqrt{\Delta^{2}+4f\;\beta \overset{¯}{T}\gamma }\right)$$

where Δ = *d*_1_ + γ + *K*_1_ − *d*_2_ − *K*_2_. Both eigenvalues are real, the positive root, λ_1_, is the dominant eigenvalue, and λ_2_ is negative. The eigenvector associated with the dominant eigenvalue is defined as

(11) $$v_{1}=\left(\frac{-\Delta +\sqrt{\Delta^{2}+4f\;\beta \overset{¯}{T}\gamma }}{2\gamma },1\right)$$

The intrinsic replication rate of the virus is now defined by the dominant eigenvalue, λ_1_, for the case where *K*_1_ = *K*_2_ = 0, i.e.,

(12) $$\rho =\frac{1}{2}\left(-\left(d_{1}+\gamma +d_{2}\right)+\sqrt{{\left(d_{1}+\gamma -d_{2}\right)}^{2}+4f\;\beta \overset{¯}{T}\gamma }\right)$$

which, apart from the constant parameters, still depends on the actual target cell density, *T̄*. This equation can be simplified by writing β in terms of ρ,

(13) $$\beta =\frac{\left(d_{1}+\gamma +\rho )(d_{2}+\rho \right)}{f\;\gamma \overset{¯}{T}}$$

which we will use later to calculate the infection rate required for obtaining a desired replication rate. Note that the same expression was obtained previously by assuming that the ratio *I*_1_(*t*)/*I*_2_(*t*) rapidly approaches a quasi–steady state (47). The downslope of the viral load during ART is defined by both eigenvalues for the case where β = 0:

(14a,b) $$\lambda_{1}=-d_{2}-K_{2}\;\text{and}\;\lambda_{2}=-d_{1}-\gamma -K_{1}$$

which are both negative.

The steady state of equation 9 implies that the determinant of the matrix equals zero, which leads to the solution

(15) $$K_{2}=\frac{f\;\beta \overset{¯}{T}\gamma }{\gamma +d_{1}+K_{1}}-d_{2}$$

(which can also be obtained by setting d*I*_1_/d*t* + d*I*_2_/d*t* = 0, or by setting λ_1_ = 0). We study the influence of CTL-mediated killing of infected target cells, *I*_1_ and *I*_2_, for three “extreme” cases: “equal” killing (*K*_1_ = *K*_2_), “early” killing (*K*_2_ = 0), and “late” killing (*K*_1_ = 0). This simplifies equation 15 into

(16a,b,c) $$K_{1}=K_{2}=\rho ,\;K_{1}=\frac{f\;\beta \overset{¯}{T}\gamma }{d_{2}}-\gamma -d_{1},\;\text{and}\;K_{2}=\frac{f\;\beta \overset{¯}{T}\gamma }{\gamma +d_{1}}-d_{2}$$

respectively. This confirms the intuition that, in the two-stage model, the total killing rate also balances viral replication. For *K*_1_ = *K*_2_ = ρ, this is obvious. For the other two cases, the *f* β*T̄*γ term is the rate at which new productively infected cells are formed per productively infected cell, which is divided by the loss rate of infected cells at the stage that is not killed, to deliver a net growth rate. This net growth rate has to be balanced by the killing and the natural loss of infected cells at the stage that is being killed.

Equation 15 can be written in terms of the replication rate, ρ, by substituting equation 13 for β:

(17a,b) $$K_{1}=\frac{\left(d_{1}+\gamma +\rho \right)\left(d_{2}+\rho \right)}{d_{2}+K_{2}}-\gamma -d_{1}\;\text{and}\;K_{2}=\frac{\left(d_{1}+\gamma +\rho \right)\left(d_{2}+\rho \right)}{\gamma +d_{1}+K_{1}}-d_{2}$$

corresponding to

(18a,b) $$K_{1}=\frac{\rho \left(d_{1}+\gamma +d_{2}+\rho \right)}{d_{2}}\;\text{and}\;K_{2}=\frac{\rho \left(d_{1}+\gamma +d_{2}+\rho \right)}{\gamma +d_{1}}$$

for the early and late killing regimes. The latter two expressions demonstrate that it is most efficient to kill during the eclipse phase, when *d*_2_ > *d*_1_ + γ. Instead, when *d*_2_ < *d*_1_ + γ, killing during the production phase, *K*_2_, requires a smaller immune response than early killing (a similar observation was made by Althaus and De Boer [32]).

### Three-stage model.

Realistic models of the HIV-1 life cycle probably require more than just two stages (48–51). The very early events after the virus has infected a target cell seem to be very important, because a large fraction of the cells dies by abortive infection (52, 53) and proteins from the incoming virus particle(s) trigger CD8^+^ T cell responses on a time scale of a few hours (54, 55). One could argue that most of the CTL-mediated killing during the eclipse phase that we considered in the two-stage model actually occurs in the first few hours after the infection of a target cell. For that reason, we here extend the model to three stages, where the first, *I*_0_, stage is thought to be short because cells rapidly enter the abortive program (at rate *d*_0_), rapidly proceed to the eclipse phase (at rate γ_0_), or are killed rapidly (at rate *K*_0_):

(19a,b) $$\frac{\text{d}T}{\text{d}t}=F\left(T\right)-\beta TI_{2},\;\frac{\text{d}E_{i}}{\text{d}t}=G\left(E_{i},V\right)-d_{E}E_{i}$$

(20) $$\frac{\text{d}I_{0}}{\text{d}t}=\beta TI_{2}-\left(d_{0}+\gamma_{0}+K_{0}\right)I_{0}$$

(21a,b) $$\frac{\text{d}I_{1}}{\text{d}t}=\gamma_{0}I_{0}-\left(d_{1}+\gamma +K_{1}\right)I_{1},\;\frac{\text{d}I_{2}}{\text{d}t}=\gamma I_{1}-\left(d_{2}+K_{2}\right)I_{2}$$

where $K_{0}=\overset{n}{\underset{i}{\sum }}k_{0_{i}}E_{i},\;K_{1}=\overset{n}{\underset{i}{\sum }}k_{1_{i}}E_{i},\;K_{2}=\overset{n}{\underset{i}{\sum }}k_{2_{i}}E_{i},$ equations 19a and b are identical to equations 7a and b, and equation 21b is identical to equation 8b. Again, *n* is the total number of immune responses, and we can set some of the killing rates to zero to allow for epitopes that are expressed at particular stages only.

The core viral replication cycle is now a linear model defined by a 3-by-3 matrix. One can again find the eigenvalues of this matrix (not shown), define the dominant eigenvalue for *K*_0_ = *K*_1_ = *K*_2_ = 0 as the effective replication rate, ρ, and write this in the same form as above, i.e.,

(22) $$\beta =\frac{\left(d_{0}+\gamma_{0}+\rho \right)\left(d_{1}+\gamma +\rho \right)\left(d_{2}+\rho \right)}{\gamma_{0}\gamma \overset{¯}{T}}$$

During ART, we set β = 0 and now find the following three eigenvalues:

(23a,b,c) $$\lambda_{1}=-d_{2}-K_{2},\;\lambda_{2}=-d_{1}-\gamma -K_{1}\;\text{and}\;\lambda_{3}=-d_{0}-\gamma_{0}-K_{0}$$

If *d*_0_ and γ_0_ are large (52, 53), this third eigenvalue will never be dominant, and the predicted downslope during ART will depend on the same two eigenvalues defined by equations 14a and b. The steady state of the linear core defined by equation 20 and equation 21 would require that

(24) $$K_{2}=\frac{\gamma_{0}\gamma \beta \overset{¯}{T}}{\left(\gamma_{0}+d_{0}+K_{0}\right)\left(\gamma +d_{1}+K_{1}\right)}-d_{2}$$

and if the early killing were the only form of killing, this would simplify into

(25) $$K_{0}=\frac{\gamma_{0}\gamma \beta \overset{¯}{T}}{d_{2}\left(\gamma +d_{1}\right)}-\gamma_{0}-d_{0}$$

Equation 24 can again be written in terms of the replication rate by substituting equation 22 to obtain

(26) $$K_{2}=\frac{\left(d_{0}+\gamma_{0}+\rho \right)\left(d_{1}+\gamma +\rho \right)\left(d_{2}+\rho \right)}{\left(d_{0}+\gamma_{0}+K_{0}\right)\left(d_{1}+\gamma +K_{1}\right)}-d_{2}$$

We add three notes to this equation. First, the scenario *K*_0_ = *K*_1_ = *K*_2_ = ρ is again a solution. Second, remember that *d*_0_ and γ_0_ are large (52, 53) and observe that *K*_0_ will have hardly any effect when *K*_0_ ≪ *d*_0_ + γ_0_. Similarly, and third, whenever (*d*_0_ + γ_0_ + ρ)/(*d*_0_ + γ_0_ + *K*_0_) ≃ 1, equation 26 will approach equation 17b.

Thus, when *d*_0_ and γ_0_ are large, the three-stage model makes predictions similar to those of the two-stage model. An alternative way to see this is to make the QSSA d*I*_0_/d*t* = 0, which seems valid because many cells are lost at an early stage (52, 53) and the early killing process is fast (54, 55), and define

(27) $$f=\frac{\gamma_{0}}{\gamma_{0}+d_{0}+K_{0}}<1$$

to observe that the γ_0_*I*_0_ term in equation 21a becomes the *fβTI*_2_ of equation 8a, which simplifies the three-stage model into the two-stage model defined above. We will first analyze the two-stage model with *f* = 1 to show that our results do not depend on having abortive infections. At the last stage, where we aim to explain recent data, we use the more-realistic three-stage model with γ_0_/(γ_0_ + *d*_0_) = 0.1, concomitantly demonstrating that our general results would also be obtained when most of the infections are abortive.

### Parameter values.

**FIG 1:**
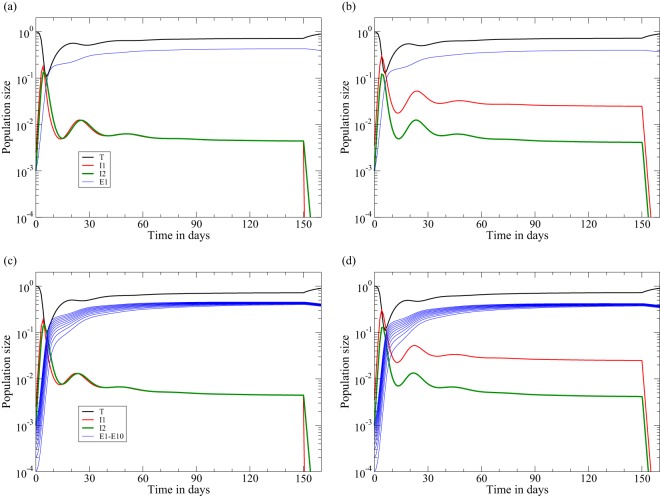
Acute infection followed by ART at day 150 in the two-stage model of equation 8. The behaviors of the early killing regime (a and c) and of the late-killing scenario (b and d) are shown. Data are shown for 1 immune response, with *k*_1_ = 10 (a and b), and 10 immune responses, with *k_i_* = 1 for *i* = 1,2,…,10 (c and d). The rate at which the viral load, *V* ∝ *I*_2_, increases during acute infection is 1.5 day^−1^ in both cases, and the rate at which it decreases during ART is approximately δ = 1 day^−1^. We use the function *F*(*T*) = *s* − *d_T_T* to complete equation 7a for the target cells and set *s* = *d_T_* to scale the uninfected steady state to one and the function *G*(*E_i_*,*V*) = *pVE_i_*/(*h_i_* + *V* + *E_i_*) with *V* = *I*_2_ to complete equation 7b of the CTL (39, 47). We here set *k*_1_ = 10 day^−1^ or *k_i_* = 1 day^−1^, *h_i_* = *i*/1,000, and *E_i_*(0) = 0.001/*i* for *i* = 1,2,…,*n*. For the early killing scenario, we set β = 8.75 and *d*_1_ = *d*_2_ = 1, and for the late-killing regime, we set β = 9.1, *d*_1_ = 0.1, and *d*_2_ = 2 day^−1^ (Table 1). By equation 13, both settings account for a realistic initial replication rate in macaques of ρ(0) = 1.5 day^−1^. See Table 1 for all other parameter values.

In the numerical simulations, we use the function *F*(*T*) = *s* – *d_T_T* to complete equation 7a for the target cells. Because not all CD4^+^ T cells are proper target cells for HIV-1 and true target cell densities are not known, we scale the maximum target cell density to 1 by setting *s* = *d_T_*. We typically set a relatively fast turnover of *s* = *d_T_* = 0.1 day^−1^ (Table 1), because activated CD4^+^ T cells are the best target cells (56) and these cells probably live for a shorter time than the average effector memory cell in HIV-1-infected patients, which have a turnover of about 0.02 per day (57, 58). The expected length of the eclipse phase is 1 day, i.e., γ = 1 day^−1^ (49, 59, 60). In the late killing regime, the model will only be consistent with the observed viral downslope of δ ≃ 1 day^−1^ when cells stay in the eclipse phase for about 1 day (see Results). Hence, the average life span of *I*_1_ cells should be more than a day, and we simply assume that they have the same expected life span of 10 days as target cells and set *d*_1_ = 0.1 day^−1^. In the early killing regime, we have to assume that cells in the eclipse phase die faster, and we set *d*_1_ = 1 day^−1^ (see Results and Table 1). The natural death rate of productively infected cells is not known (61), and we set it to *d*_2_ = 1 day^−1^ in the early killing regime and to *d*_2_ = 2 day^−1^ in the late killing scenario (see Results and Table 1). In the three-stage model, we take into account that most infected cells die rapidly by abortive infection (52, 53) and set *d*_0_ = 54 and γ_0_ = 6 day^−1^, such that *f* = 0.1, and in the two-stage model we set *f* = 1 for reasons of simplicity (which does affect the dynamics, but not our conclusions). The infection rate β is parameterized by requiring an initial replication rate ρ(0) = 1.5 day^−1^, using equation 13. In macaques, CD8^+^ effector T cells proliferate at a rate of one per day (44) and have an expected life span of several months to a year (62, 63). We give them an expected life span of 100 days, *d_E_* = 0.01 day^−1^, and correspondingly, set *p* = 1.01 day^−1^ (Table 1). Using the function

(28) $$G\left(E_{i},V\right)=\frac{pVE_{i}}{h_{i}+V+E_{i}}$$

with *V* = *I*_2_ to complete equation 7b of the CTL (39, 47), we have tuned the saturation constants, *h_i_*, such that the immune responses approach their steady state in a few weeks (Fig. 1). For the low saturation constants that we use, the steady-state immune response of equation 7b, i.e., *Ē_i_* = *V̄*(*p*/*d_E_* − 1) − *h_i_* = 100*V̄* − *h_i_*, implies that the magnitude of each individual immune response is more or less proportional to the viral load, *V̄*, and fairly independent of its saturation constant, *h_i_*, whenever *h_i_* < 100*V̄* (47).

## RESULTS

### Downslopes of the viral load during ART.

In monkeys and patients treated with potent ART, the viral load in the peripheral blood decreases at a rate of δ ≃ 1 day^−1^ (30) for about a week. The mere fact that monkeys that were treated with ART following the depletion of their CD8^+^ T cells also had downslopes of δ ≃ 1 day^−1^ seems difficult to reconcile with the notion that the major protective effect of CTL is cytolytic in the one-stage model (11, 12). Two decades ago, Klenerman et al. (31) developed a model where the HIV-1-infected cells progress through several stages before they commence to produce viral particles (i.e., a model similar to equation 8) and demonstrated that the slope, δ, with which the viral load declines during ART is defined by the slowest time scale of the various stages of infected cells. Since we now know that the average length of the eclipse phase, 1/γ in our model, is about 1 day (49, 59, 60), their result is in excellent agreement with the general observation that δ ≃ 1 day^−1^ (30), that δ is independent of the viral load and CD4^+^ T cell count (29), and that δ remains similar in CD8^+^ T cell-depleted monkeys (11, 12).

Fifteen years later Althaus and De Boer (32) extended these results by showing that rapid killing during the early stage (*K*_1_ ≫ *K*_2_) is also compatible with all these observations if the expected life span of productively infected cells, 1/*d*_2_, is the slowest time scale of the model. The invariant downslope, δ, should then reflect the intrinsic death rate of productively infected cells, implying that *d*_2_ ≃ 1 and *K*_2_ ≪ *d*_2_. Rapid killing at the early stage could be due to the immune responses to proteins of the incoming virus (54, 55), and slow killing at the late stage could be due to downregulation of MHC expression by the late expression of Nef (32). Importantly, both killing regimes imply that the downslope, δ, during ART fails to provide information on the rate at which infected cells are killed (31, 32).

Both results are readily confirmed by equations 14a and b, providing the two negative eigenvalues of equation 10 for β = 0 and illustrating that the observed downslope of the viral load, *V*(*t*), during ART will be δ ≃ *d*_1_ + γ + *K*_1_ whenever |λ_1_| ≫ |λ_2_|, whereas the downslope will reflect δ ≃ *d*_2_ + *K*_2_ whenever |λ_1_| ≪ |λ_2_|. For simplicity, consider the three cases of equal killing, early killing, and late killing, i.e., *K* = *K*_1_ = *K*_2_, *K*_2_ → 0, and *K*_1_ → 0, respectively. For equal killing, i.e., |λ_1_| = *d*_2_ + *K* and |λ_2_| = *d*_1_ + γ + *K*, we would obtain either that δ ≃ *d*_1_ + γ + *K* or that δ ≃ *d*_2_ + *K*, and since both slopes depend on the killing rate, this is not in agreement with the observation that the downslope is unaffected by the depletion of CD8^+^ T cells (11, 12). Thus, a two-stage model with similar killing rates at both stages is—like the one-stage model—not compatible with the data (if CTL are considered to be killers).

Next consider late killing (31) by setting *K*_1_ = 0. Whenever *d*_1_ + γ ≪ *d*_2_ + *K*_2_, the observed downslope of the viral load, *V*(*t*), during ART will be δ ≃ *d*_1_ + γ, which is independent of the killing rate and would be in agreement with the general observation δ ≃ 1 day^−1^ (11, 12, 29, 30) when *d*_1_ + γ ≃ 1 day^−1^. Since δ ≃ 1 after CD8^+^ T cell depletion (11, 12), this late killing regime requires that *d*_1_ + γ ≪ *d*_2_ (which we obtain by setting *d*_2_ = 2 day^−1^, implying fairly rapid death of cells that produce virus; Table 1). Finally, consider early killing (32) by setting *K*_2_ = 0. Whenever *d*_1_ + γ ≫ *d*_2_, we obtain that δ ≃ *d*_2_. To be consistent with all data, this early killing regime therefore requires *d*_2_ ≃ 1 day^−1^, again implying rapid death of productively infected cells, and to be consistent with the CD8^+^ T cell depletion, we require *d*_1_ + γ > 1 day^−1^, suggesting fairly rapid death during the eclipse phase (52, 53). We realized this by setting *d*_1_ = *d*_2_ = 1 day^−1^ (Table 1).

A numerical confirmation of these results is depicted in Fig. 1, where an infection with *n* = 1 or *n* = 10 immune responses is treated with perfect ART (i.e., we set β = 0 at day 150). Although the killing rates at the pretreatment steady state are relatively fast and somewhat different, i.e., *K*_1_ ≃ 4.3 day^−1^ (Fig. 1a), *K*_2_ ≃ 4 day^−1^ (Fig. 1b), *K*_1_ ≃ 6.3 day^−1^ (Fig. 1c), and *K*_2_ ≃ 5.8 day^−1^ (Fig. 1d), the downslope of the viral load [here *I*_2_(*t*)] reflects the rate at which productively infected cells die, δ ≃ *d*_2_ = 1, in Fig. 1a and c, whereas it reflects the rate at which the early infected cells, *I*_1_(*t*), depart from the eclipse phase, i.e., δ ≃ *d*_1_ + γ ≃ 1.1 day^−1^, in Fig. 1b and d. If one were to add lines with an exponential downslope of 1 per day in Fig. 1a and c, or 1.1 per day in Fig. 1b and d, these lines would almost perfectly coincide with the straight green lines depicting the downslope of the viral load (*I*_2_) from day 150 onwards. Thus, the downslopes in the model are in excellent agreement with the observed downslopes of the viral load. Note that the 10 immune responses approach similar magnitudes (see the subsection on parameter values above) and that 10 independent immune responses with a 10-fold-lower killing rate, *k_i_* = 1, control somewhat better than a single response with *k*_1_ = 10 (this is due to the absence of direct competition between clones and the presence of intraspecific competition among CTL of the same specificity [39]). Finally note that the ratio *I*_1_/*I*_2_ is about 1 in the early killing regime and larger than 1 in the late killing regime, which can be understood from the killing of *I*_1_ cells in the early regime and of *I*_2_ cells in the late killing scenario.

To summarize, the downslopes of the viral load during ART are not expected to provide reliable information on the rate at which productively infected cells are killed by CD8^+^ T cells (26, 31, 32). Rapid killing is expected to be masked by the slower phases of the viral life cycle.

### Downslope after the peak viral load.

Acute infections with HIV-1, simian immunodeficiency virus (SIV), and simian-human immunodeficiency virus (SHIV) are characterized by an initial phase of rapid viral replication [at rate ρ(0)] that ends with a peak viral load, which is followed by a phase during which the viral load declines fairly rapidly until it slowly approaches the viral set point (Fig. 1). The downslope following the peak (sometimes called α [40]) is typically estimated to vary around α = 1 day^−1^ (33, 35, 40) and, in vaccinated monkeys, is not significantly affected by the presence of a large cellular immune response (33, 34). The maximum downslope that can be obtained in our model is achieved when target cells are completely depleted and the immune responses are maximal. Since setting *T* = 0 in equation 10 is the same as setting β = 0, we can reuse equations 14a and b to predict the maximum downslope that would be approached shortly after complete target cell depletion. Similar to the situation after the onset of ART, in the early and late killing regime, the maximum downslope following the peak viral load is not expected to depend on the killing rates, *K*_1_ or *K*_2_, and would be *d*_2_ or *d*_1_ + γ, respectively. Thus, it is not surprising that similar downslopes were obtained in control and vaccinated monkeys (47), and an eclipse phase of about 1 day (49, 59, 60) would readily explain that the observed downslopes are less than α = 1 (47).

Petravic and Davenport (35) have studied the peak viral load, the nadir of CD4^+^ T cell numbers, and the downslope, α, of the viral load after the peak in macaques acutely infected with CXCR4-tropic SHIV (which should be able to infect all CD4^+^ T cells). Combining data from unvaccinated and vaccinated monkeys, they report a negative correlation between the nadir of the CD4^+^ T cells and the peak viral load and a positive correlation between the peak viral load and the downslope α. Because high viral loads are expected to reduce target cell numbers, the negative correlation seems a natural result (which nevertheless was not confirmed in a recent human study [41], probably because CD4^+^ T cell numbers are a poor measure of target cell availability for CCR5-tropic HIV-1). The positive correlation is more interesting because high viral loads should be associated with poor immune control and, hence, a longer life span of productively infected cells and not with a faster downslope. This positive correlation has therefore been used as evidence against cytolytic control by the CD8^+^ T cells (35). A positive correlation is indeed expected when the cellular immune response is not affecting the life span of productively infected cells, because a good immune response should be associated with a higher nadir of the target cells, enabling faster replication of the virus (35). By mathematical modeling, Petravic and Davenport (35) show that the correlation between the peak viral load and the downslope, α, should always be positive in models where the cellular immune control is nonlytic, whereas this relation should be nonmonotonic in models where the CTL are cytolytic. In the latter case, the correlation will be negative when the target cells are severely depleted, because then the downslope, α, reflects the death rate, δ, of productively infected cells. Although Petravic and Davenport (35) demonstrate that a strictly positive correlation is also expected when infected cells are killed during the eclipse phase (i.e., our early killing regime), they nonetheless suggest that vaccine-induced CD8^+^ T cells control SHIV infection by noncytolytic means.

**FIG 2:**
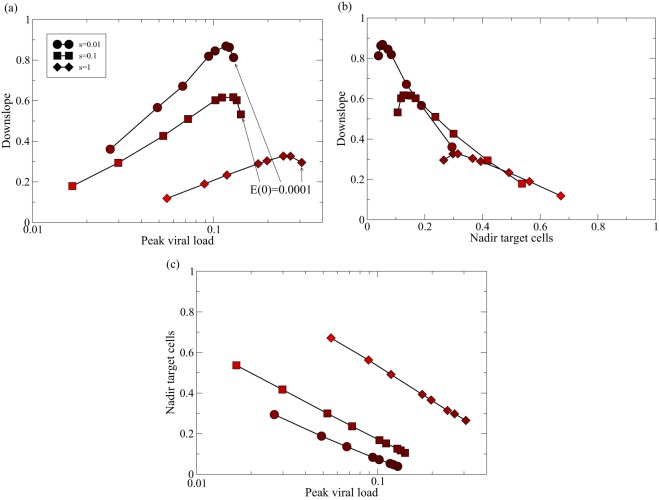
Relationships between the peak viral load, nadir of target cells, and downslope of the viral load after the peak. To mimic the effect of vaccination, we study acute infection in the late killing regime (as in Fig. 1b, with *n* = 1, *h*_1_ = 0.001, and *k*_1_ = 10), now varying the initial number of effector cells from *E*(0) = 0.0001, 0.0005, 0.001, 0.005, … , 0.01, 0.05, 0.1, 0.2, 0.3, 0.4 to *E*(0) = 0.5. These initial conditions can be recognized in the figure because the peak viral load (i.e., I_2_max) decreases with the E(0) (a, arrows) and the nadir of the target cells (i.e., *T*_min_) increases with *E*(0). During the first 2 weeks of the infection, we record the peak viral load and the nadir of the target cells, and we estimate the downslope of the viral load after the peak by linear regression on the natural logarithms of the *I*_2_ values (over the first 4 days following the peak). We repeat this procedure for different turnover rates of the target cells (i.e., *s* = *d_T_* = 0.01, 0.1, and 1 day^−1^; see the key). The darkness of the symbols depicts the nadir of the target cells (where bright means *T*_min_ = 1 and black means *T*_min_ = 0; in panels b and c, the nadir can also be read from the *x* or *y* axis). (a) The correlation between the peak viral load and the downslope of the viral load after the peak is positive over a domain of peak viral load values [and is only negative for *E*(0) ≤ 0.001, i.e., in “unvaccinated” systems]. (b) The correlation between the nadir of the target cells and the downslope is largely negative, which was to be expected because higher availability of target cells decreases the downslope. (c) The peak viral load and the nadir of the target cells are negatively correlated. Similar results are obtained for *k* = 1 and *k* = 100 (not shown).

We have seen in the discussion above that, even in the late killing regime, the downslope, α, need not represent the rate at which productively infected cells are killed and, instead, should reflect the time cells spend in the eclipse phase. We therefore repeat the analysis of Petravic and Davenport (35) for the *in silico* acute infection depicted in Fig. 1b. To account for vaccination, we vary the number of effector cells, *E*(0), at the onset of the infection and record the peak viral load, nadir of the target cells, and the downslope of the viral load after the peak (Fig. 2). These results agree with the observed negative correlation between the nadir of the target cells and the peak in the viral load (Fig. 2c) and the predicted nonmonotonic relation between the downslope and the peak viral load (Fig. 2a). Note that the downslope is largely determined by the availability of target cells, as the data for different rates of target cell turnover, *s* = *d_T_*, more or less fall on the same line in Fig. 2b. Although the relation between peak viral load and the downslope is nonmonotonic in our model, the region where this correlation is negative is small (on a log scale), has little effect on the downslope, and is confined to cases with low initial numbers of effector cells, i.e., *E*(0) ≤ 0.001 (Fig. 2a). This suggests that the correlation should be positive among vaccinated animals and when vaccinated and control animals are mixed but should be small or ambiguous in control animals (with severe target cell depletion and high downslopes). We reanalyzed the data in the study of Petravic and Davenport (35) by performing the same Spearman correlations on these subsets of the data and confirmed this prediction; i.e., *r* = 0.5 with *P* = 0.03 in vaccinated monkeys and *r* = 0.29 with *P* = 0.31 in control animals. To summarize, the two-stage model with late killing also accounts for an overall positive correlation between peak viral load and the downslope, α, and observing a positive correlation provides little evidence on how CD8^+^ T cells control SHIV infection.

Similarly, the fact that wild-type SHIV and immune escape SHIV mutants decline at similar rates following the peak viral load (20) should not be used as evidence against cytolytic control. Even if cells productively infected with wild-type SHIV are killed much faster than those infected by a mutant that has escaped from a cytolytic CD8^+^ T cell response, their corresponding viral load would have a very similar rate of decline when the killing is either largely early or largely late (equations 14a and b).

### Upslopes of the viral loads.

Given the fact that the downslopes of the two-stage model cannot be used to estimate killing rates, we now turn to the upslopes of the viral loads observed during acute infection, following CD8^+^ T cell depletion (11, 12), and during immune escape (36). The rate at which the viral load increases in the two-stage model of equation 8 is determined by the dominant eigenvalue defined in equation 10, which depends on the availability of target cells, *T̄*, and the total killing rates, *K*_1_ and *K*_2_.

### (i) Viral replication during the acute phase of the infection.

The initial rate of expansion of the viral load during the acute phase of the infection before the onset of immune responses is defined by ρ(0), i.e., equation 12 with *T̄* = *T*(0). In Materials and Methods, we estimate that ρ(0) ≃ 1 day^−1^ in humans (40, 41) and that ρ(0) ≃ 1.5 day^−1^ in macaques (34, 43–45). Equation 13 allows us to define the effective infection rate, β, delivering the required expansion rate (47). For instance, to obtain the observed expansion rate in macaques of ρ(0) ≃ 1.5 day^−1^ in the late-killing model, we substitute its parameters *d*_1_ = 0.1, *d*_2_ = 2, and γ = 1 into equation 13 to find that β = 9.1/[*fT*(0)] = 9.1 day^−1^ [because *fT*(0) = 1]. Similarly, for the early killing model, with *d*_1_ = *d*_2_ = γ = 1, we find that β = 8.75/[*fT*(0)] = 8.75 day^−1^. Since the eigenvector associated with the dominant eigenvalue defines the ratio *I*_1_(*t*)/*I*_2_(*t*), we can also substitute *T̄* = *T*(0) and *K*_1_ = *K*_2_ = 0 into equation 11 and use the same two sets of parameters to obtain, in the acute phase of the infection, the ratios *I*_1_(*t*)/*I*_2_(*t*) = 2.5 and *I*_1_(*t*)/*I*_2_(*t*) = 3.5 for the early and late killing models, respectively. The four numerical examples depicted in Fig. 1 indeed have an initial expansion rate of ρ ≃ 1.5 day^−1^ until the viral load approaches its peak value.

### (ii) Viral replication during the chronic phase of the infection.

The effective replication rate, ρ, during chronic infection will not be the same as the ρ(0) computed above, because the availability of target cells, *T̄*, is no longer defined by *T*(0). *T̄* could be lower, due to target cell depletion, or higher, due to immune activation. The chronic viral replication rate can be estimated by a perturbation of the chronic steady state, setting the CTL killing rate to 0, which has been achieved in several experiments by depleting the CD8^+^ T cells (9–15). The viral load increases steeply in these experiments over a period of several days, and in all three models, this slope should reflect the effective replication rate, ρ, during the chronic steady state. In the Appendix, we perform a meta-analysis of these data to estimate the rate at which the viral load increases. We find that, typically, ρ ≃ 0.5 day^−1^ (9–15) and that ρ ≃ 1 day^−1^ in monkeys with very low viral loads (13–15).

### (iii) Estimating the killing rates from the observed effective replication rate.

**FIG 3:**
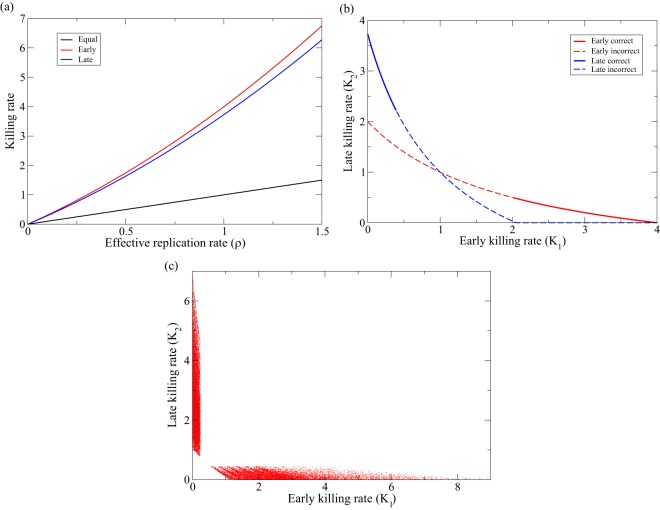
Killing rates predicted by equation 17. (a) The red line depicts *K*_1_ for *K*_2_ = 0 as a function of ρ, the blue line *K*_2_ for *K*_1_ = 0 (see equations 18a and b, respectively), and the black line is just the line *K* = *K*_1_ = *K*_2_ = ρ. (b) *K*_2_ is depicted as a function of *K*_1_ for ρ = 1, using equation 17b for the early killing parameters, *d*_1_ = γ = *d*_2_ = 1 (in red), and for the late killing parameters, *d*_1_ = 0.1, γ = 1, and *d*_2_ = 2 day^−1^ (in blue). Note that we indeed predict that the lines cross at *K*_1_ = *K*_2_ = ρ = 1. The lines are solid when 0.5 < δ < 1.5 and dashed otherwise. (c) We make small steps through the parameter space for 0.01 ≤ *d*_1_, *d*_2_ ≤ 5, and 0.5 ≤ ρ ≤ 1, drawing a random value for either *K*_1_ or *K*_2_ from a uniform distribution, 0 < *K*_1_, *K*_2_ < 10, and use equation 17a or b to compute the other killing rate. This analysis is performed for γ = 1, and we increase the parameters by 10% when we step through the parameter space. Here, we are more strict and only accept solutions when 0.75 < δ < 1.25 (in the presence and absence of CTL).

Knowing that during chronic infection, 0.5 ≤ ρ ≤ 1 day^−1^, we can use equation 17 to compute the killing rates that are required to keep the infection at steady state (Fig. 3). For the maximum effective replication rate of ρ = 1 day^−1^, equation 17 would predict killing rates of *K*_1_ = *K*_2_ = 1, *K*_1_ = 4, and *K*_2_ = 3.7 day^−1^, for the equal, early, and late killing regimes, respectively (Fig. 3a). For the minimum replication rate, ρ = 0.5 day^−1^, these would be *K*_1_ = *K*_2_ = 0.5, *K*_1_ = 1.75, and *K*_2_ = 1.6 day^−1^, respectively.

Equation 17 enables us to compute the early and late killing rates for any value of the effective replication rate (Fig. 3b). However, not every combination of *K*_1_ and *K*_2_ will be in agreement with the observed downslope, δ = 1 day^−1^, during ART (32). Since δ is defined by the dominant eigenvalue of equations 14a and b, i.e., δ = min(|λ_1_|, |λ_2_|), we can check which combinations of *K*_1_ and *K*_2_ obey the observation δ ≃ 1. Points obeying the condition 0.5 < δ < 1.5 are therefore depicted by the heavy lines in Fig. 3b, and all “incorrect” points on the lines defined by equations 17a and b are shown as dashed lines. We observe that the combination of the condition that δ ≃ 1 during ART (in the presence and absence of CTL) and the highest observed effective replication rate, ρ ≃ 1 day^−1^, strongly constrains the estimated killing rates. First, the killing has to be either mostly early or largely late, as similar killing rates fail to satisfy the δ ≃ 1 condition. Second, we see that for ρ = 1 day^−1^, the required killing rates are fairly large, i.e., *K*_1_ > 2 day^−1^ and *K*_2_ > 2.3 (Fig. 3b).

We perform a similar analysis by stepping through the parameter space with small increments for *d*_1_, *d*_2_, and ρ, drawing a random value for either *K*_1_ or *K*_2_ from a uniform distribution, 0 < *K*_1_, *K*_2_ < 10, and using equation 17 to compute the other killing rate (Fig. 3c). Drawing 2.6 × 10^6^ parameter combinations, we find that about 1% of these are in agreement, with a slope of 0.75 < δ < 1.25 during ART (in the presence and absence of CTL). The fact that most symbols are located along the axes of Fig. 3c shows that the killing rates, *K*_1_ and *K*_2_, cannot both be high when one selects points in agreement with the observed downslopes, In the early and late killing regimes, the killing rates can be quite high, i.e., 1 < *K*_1_, *K*_2_ < 6 day^−1^ (Fig. 3c). Note that equation 17 is independent of the functions *F*(*T*) and *G*(*E_i_*,*V*) and that these estimates should therefore be relatively generic. Finally, the highest killing rates in Fig. 3c correspond to the parameter settings in which the virus is replicating fast (large ρ) and target cells are dying fast (large *d*_1_ and/or *d*_2_), such that the window of opportunity for the CTL to kill a target cell is short. Since we require the behavior of the model to be consistent with the observed downslopes, such high killing rates cannot be excluded. However, if the eclipse phase and the production phase both take about a day, the killing rates should vary between 1.5 < *K*_1_, *K*_2_ < 4 day^−1^ (Fig. 3a and b).

### (iv) Revisiting the upslope following CD8 depletion.

Elemans et al. (22) analyzed the data from CD8 depletion experiments with a variety of models, arguing that the initial rate of increase of viral load reflects the rate of killing prior to the removal of the CTL. For a single-stage infection, this is true, but for the two-stage infection model, this is more complicated. For the late-stage killing scenario, one readily observes from the steady state of d*I*_2_/d*t* in equation 8 that γ*I*_1_ − *d*_2_*I*_2_ = *K*_2_*I*_2_ before CD8^+^ T cell depletion and, hence, that d*I*_2_/d*t* = γ*I*_1_ − *d*_2_*I*_2_ = *K*_2_*I*_2_ immediately afterwards. Thus, in the late-stage killing scenario, the initial upslope of the viral load is indeed expected to reflect the total killing rate. For the early-stage killing scenario, one solves from d*I*_1_/d*t* = 0 that *Ī*_1_ = *f*β*TI*_2_/(γ + *d*_1_ + *K*_1_). Substitution, and setting *K*_1_ = 0 to account for the CD8 depletion, give

(29) $$\frac{\text{d}I_{2}}{\text{d}t}=\left[\frac{f\;\beta T\gamma }{\gamma +d_{1}}-d_{2}\right]I_{2},$$

which defines a growth rate that is not equal to *K*_1_ (equation 16b) and not equal to ρ (equation 18a). Actually, the term between the brackets is equal to *K*_2_ defined by equation 16c, showing that the upslope in the early killing regime reflects what the killing rate would have been if the killing were “late only.”

**FIG 4:**
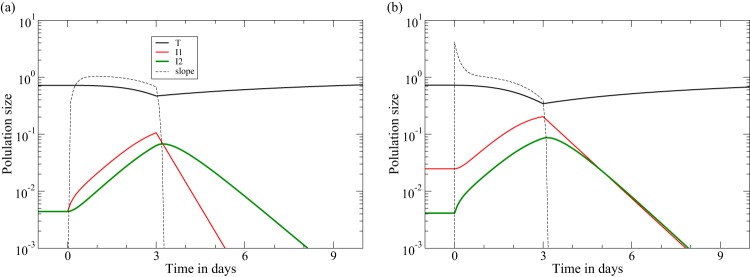
CD8^+^ T cell depletion at day zero is followed by ART at day three (11, 12). We start the model in the chronic states from Fig. 1 (using the same functions and parameters). The dashed line provides the rate of increase of the productively infected cells, d*I*_2_/d*t*/*I*_2_, and confirms that the initial rate at which the viral load increases reflects the steady-state killing rate in the late killing regime (*K* = 4.0) (b) but not in the early killing regime (a). In both regimes, the rate at which the viral load increases rapidly approaches the effective replication rate of ρ ≃ 1 day^−1^, until the target cell numbers decline. Due to our design of the parameters *d*_1_ and *d*_2_, the downslope of the viral load during ART remains δ ≃ 1 day^−1^ in both regimes (see Table 1).

However, in a two-stage model, the upslope is defined by two eigenvalues, defining both an initial transient and the ultimate upslope defined by the dominant eigenvalue. The two upslopes calculated above define the short initial transient before the upslope approaches the dominant eigenvalue, ρ. The characteristic length of this initial transient is determined by the negative eigenvalue of equation 10, and at time zero in Fig. 4a and b, we compute numerically that λ_2_ = −4.2 and λ_2_ = −4.4 day^−1^, respectively. The numerical simulations in Fig. 4 confirm this and show that the regime where the viral load increases at a rate of ρ ≃ 1 day^−1^ is approached in about half a day and lasts for a few days, until the target cells levels start dropping. The rate at which the viral load increases over a period of a few days will therefore not reflect the killing rate but will rapidly approach the replication rate, ρ, in the chronic steady state. Fortuitously, this means that the CD8 depletion data provide estimates for ρ (see the meta-analysis in the Appendix). The simulations depicted in Fig. 4 look very similar to the CD8 depletion experiments fitted by Elemans et al. (22). We have previously fitted the two-stage model to the same data sets and found that the model can describe the data well for a wide range of parameter values (26, 64). Statistically speaking, fitting such a complicated model to such sparse data is not informative because most parameters turned out to be unidentifiable (not shown).

### Estimating killing rates from the observed immune escape rates.

HIV-1 readily escapes from CD8^+^ T cell immune responses by mutations in the major histocompatibility complex (MHC) class I restricted epitopes. The rate at which viral variants carrying one or several immune escape mutations take over the viral quasispecies has been used to estimate the selective pressure imposed by the immune response the virus is escaping from (reviewed by Regoes et al. [65]). These replacements are typically fast during acute infection (6, 7, 66) but tend to be slow during chronic infection (8, 36, 67). Since in our model, the immune responses approach a similar magnitude (Fig. 1), the killing rate of a viral variant that just escaped one immune response is approximately the fraction (*n* − 1)/*n* of that of the wild-type virus. The initial selection coefficient of such a variant is defined by its replication rate in the chronic steady state that is still largely determined by the wild-type virus experiencing *n* immune responses. Since the replication rate is defined by the dominant eigenvalue of equation 10, we again use equation 13 to replace the *f*β*T̄*γ term and obtain for the effective replication rate of a viral strain escaping one immune response during chronic infection

(30) $$\lambda \prime =\frac{1}{2}\left(-\left(d_{1}+\gamma +K_{1}\prime +d_{2}+K_{2}\prime \right)+\sqrt{{\left(d_{1}+\gamma +K_{1}\prime -d_{2}-K_{2}\prime \right)}^{2}+4\left(d_{2}+\rho \right)\left(d_{1}+\gamma +\rho \right)}\right)$$

where $K_{1}\prime =\frac{n-1}{n}K_{1}\;\text{and}\;K_{2}\prime =\frac{n-1}{n}K_{2}\;$ with *K*_1_ and *K*_2_ defined by equations 18a and b (if the epitope was expressed both early and late).

**FIG 5:**
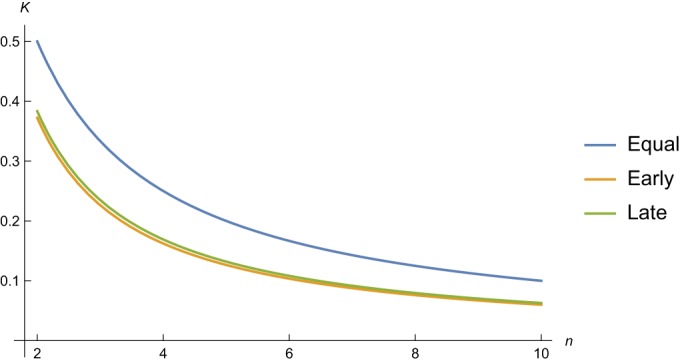
Initial rate at which a mutant escaping one immune response takes over the viral quasispecies. We use equation 30 to predict the escape rate as a function of the breadth of the immune response, *n*, for a wild-type replication rate of ρ = 1 day^−1^. Note that these results are independent of the functions *F*(*T*) and *G*(*E_i_*,*V*) in equations 7a and b. The parameters are as described in Table 1.

The initial expansion of the mutant obeys *M*(*t*) = *M*(0)e^λ′*t*^, and since the wild type virus initially remains at steady state (i.e., λ ≃ 0), λ′ provides the rate at which the mutant replaces the wild type, i.e., the escape rate. This means that we can predict the escape rate as a function of ρ, and the breadth of the immune response *n*, in a manner that is independent of the form of the functions *F*(*T*) and *G*(*E_i_*,*V*) (Fig. 5). Even for a rapid effective replication rate requiring a strong immune response, e.g., ρ = 1 requiring *K*_1_ = 4 or *K*_2_ = 3.72 day^−1^ (Fig. 3), we predict escape rates around λ′ = 0.1 day^−1^ for immune responses with a breadth of *n* > 5 in both the early and late killing regimes (Fig. 5). Unlike the simple λ′ ≃ ρ/*n* = *K*/*n* of the single-stage model (equation 6), with equation 30, we obtain escape rates that are much smaller than *K*_1_/*n* or *K*_2_/*n* and even somewhat smaller than ρ/*n* (Fig. 5). Thus, slow immune escapes are perfectly consistent with rapid killing rates because the escape rate is not reflecting the breadth-weighted killing rate, *K*/*n*, but on average defines a lower bound on the breadth-weighted replication rate, ρ/*n*. Given that viral replication is fairly slow, i.e., 0.5 < ρ < 1 day^−1^, it is no longer surprising that immune escape rates observed during the chronic phase of the infection tend to be slow. Note that we have previously argued that estimating the killing rate by a combination of immune escape and reversion data also depends on the viral replication rate (68) and that rapid viral replication during reversion may lead to an overestimation of the killing rate at a later phase with slower replication. These problems do not apply here because we consider the steady state of a chronic infection.

### Three-stage model.

Chowdhury et al. (15) demonstrate that the upslope of the viral load following CD8^+^ T cell depletion is about twofold faster in controller monkeys with low viral loads than in “progressor” monkeys with high viral loads (i.e., ρ ≃ 1 and ρ ≃ 0.5 day^−1^, respectively; see the Appendix). They suggest that the CD8^+^ T cells exert a better control in monkeys with a low viral load (15). Our results confirm this because a better immune control, e.g., a killing rate, *K*, should indeed correspond to faster viral replication (equation 18). In our model, this faster viral replication is realized by a better preservation of target cells when the killing is faster (Fig. 1). Our results therefore suggest that target cell levels should be higher in controllers than in progressors, which at least correlates well with the higher CD4^+^ T cell counts in controllers (1, 15).

**FIG 6:**
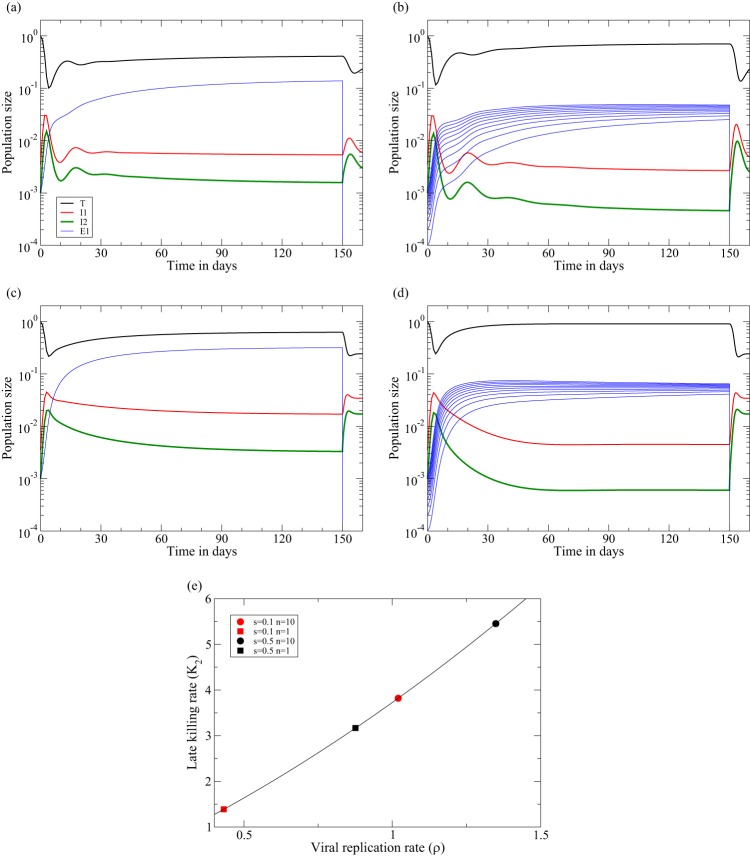
Examples of progressor and controller monkeys simulated with the quasi–steady-state version of the three-stage model of equation 21. Progressors are made by giving them just one immune response (with k_2_1__ = 10), whereas controllers are given 10 immune responses (with k_2_i__ = 10 for *i* = 1,2,…,10), Performing CD8^+^ T cell depletion at day 150, we confirm the findings of Chowdhury et al. (15), showing that the viral load increases faster in monkeys that control better. (a) *s* = *d_T_* = 0.1, *n* = 1: ρ = 0.43 and *K*_2_ = 1.4 day^−1^. (b) *s* = *d_T_* = 0.1, *n* = 10: ρ = 1.02 and *K*_2_ = 3.8 day^−1^. (c) *s* = *d_T_* = 0.5, *n* = 1: ρ = 0.88 and *K*_2_ = 3.2 day^−1^. (d) *s* = *d_T_* = 0.5, *n* = 10: ρ = 1.35 and *K*_2_ = 5.4 day^−1^. (e) Confirmation that all combinations fall on the line predicted by equation 18b. Parameters are for the late killing regime with k_2_i__ = 10, and *h_i_* = *i*/1,000 for *i* = 1,2,…,*n*, β = 91, *d*_0_ = 54, and γ_0_ = 6 day^−1^.

To confirm these results in a quantitative manner, we simulate these CD8^+^ T cell depletion experiments in our most realistic three-stage model, where we allow most CD4^+^ T cells to die from abortive infection (52, 53). Setting *d*_0_ = 54 day^−1^ and γ_0_ = 6 day^−1^, we let about 10% of cells that become infected survive into the eclipse phase, in about 4 h [i.e., *f* = γ_0_/(γ_0_ + *d*_0_) = 0.1 in equation 27]. Interestingly, this leads to a steady state with realistically low fractions of infected CD4^+^ T cells (Fig. 6) (69, 70). We make controller and progressor monkeys by varying the breadth of the immune response (Fig. 6) and pick a regime where most of the killing is late (early killing gives similar results [not shown]). Making a progressor monkey by allowing for just 1 immune response, we obtain a realistic upslope, following CD8^+^ T cell depletion, of ρ = 0.43 day^−1^ with a killing rate of *K*_2_ = 1.4 day^−1^ (Fig. 6a). A controller monkey with *n* = 10 immune responses approaches a replication rate of ρ = 1.02 day^−1^ (with a killing rate of *K*_2_ = 3.8 day^−1^) and, indeed, has higher target cell availability (Fig. 6b) than the progressor monkey in Fig. 6a. In addition to increasing the viral replication rate by elevating the killing rate, we can also increase ρ more directly by increasing the rate of target cell production, *s*. A fivefold increase in target cell production indeed increases both the replication rate and the killing rate (Fig. 6c and d).

Note that all four combinations of ρ and *K*_2_ in Fig. 6 fall on the line predicted by equation 18b, suggesting an almost linear increase of the killing rate with the replication rate. In combination, these results predict that patients with a high production of target cells should have fast viral replication and high viral loads with rapid killing, whereas patients with a broad immune response should have low viral loads and high target cell levels and, hence, also have rapid viral replication (see the Appendix for a generalization of these results). Finally, note that the viral set point is approached monotonically in Fig. 6c and d, which is realistic (40, 41) but difficult to achieve with this type of model (47).

## DISCUSSION

Assuming that the major protective effect of CTL during HIV-1 infection is to kill infected cells fast, and considering mathematical models with an eclipse phase, we have shown that the total killing rate has to be considerably faster than one per day and that the killing rate should vary over the viral life cycle. Since the killing rate has to balance the viral replication rate, we were able to estimate the killing rate from the observed upslope of the viral load following CD8^+^ T cell depletion. Because alternative models in which the major protective effect of CTL is nonlytic are also consistent with all data (11, 22, 24), we obviously cannot prove that the killing rates are this fast. Our main result is to demonstrate that the current data provide no evidence that the killing rate is slow (in contrast to the intuitive interpretation that they do).

The killing rates that we estimate for SIV infection in macaques, i.e., 1.6 < *K_i_* < 4 day^−1^, remain conservative. For instance, if virus-producing cells were to die (or burst) more rapidly than we assumed for the late killing parameters (i.e., in about half a day, *d*_2_ = 2 day^−1^), the corresponding killing rate, *K*_2_, should be even larger to balance the effective viral replication (equation 18b). Similarly, if killing during the first few hours after the infection of a cell would indeed be important (32, 54, 55), it would require a killing rate, *K*_0_, that is at least an order of magnitude larger than the 4 day^−1^ estimated above (equation 25; also references 52 and 53). The predicted killing rates in patients infected with HIV-1 could be somewhat lower, however, because the initial replication rates of the virus in humans [ρ(0) ≃ 1 day^−1^ (40, 41)] are estimated to be lower than those in monkeys [ρ(0) ≃ 1.5 day^−1^ (34, 43–45)]. A rough estimate for the rate of viral replication during the chronic phase in patients would be that this is also about one third lower than that in monkeys, i.e., 0.3 < ρ < 0.7 day^−1^. Because the relation between the required rate of killing and the rate of viral replication is almost linear (Fig. 6E), this would mean that the killing rates in humans should be about one-third lower than those reported in Results.

In a recent paper, Halle et al. employed two-photon microscopy in mice to enumerate the number of virus-infected target cells that were killed by CTL per day (71). Using transgenic and normal CTL, they estimated that one CTL kills 2 to 16 target cells per day (71). This estimate of a few dead targets per CTL per day is very similar to our previous estimates on the *in vivo* killing of splenocytes pulsed with lymphocytic choriomeningitis virus (LCMV) epitopes (72). An HIV-1-infected patient having about 1% cognate CTL in a total pool of approximately 5 × 10^10^ CD8^+^ T cells would therefore be able to kill about 10^9^ HIV-1-infected cells per day. Since the total number of productively infected cells is estimated to be 10^8^ cells (70, 73) and these cells have an expected life span of about 1 day (30), it seems perfectly feasible that most productively infected cells die from cytotoxic activity.

In our models, the killing rate increases only marginally if the breadth, *n*, or the quality, *k_i_*, of the immune response is increased (Fig. 1 and 6). An intuitive explanation for this is that the total killing rate ultimately has to balance the viral replication rate, which largely depends on the availability of target cells. Whenever target cell availability is high and the viral replication rate approaches its maximum [ρ → ρ(0)], any further increases in breadth or quality hardly increase the killing rate (38, 39), and each immune response would on average contribute a killing rate of less than ρ(0)/*n* (Fig. 5). Increasing the breadth would therefore hardly improve the control of the viral load but would provide much better resistance against the evolution of immune escapes, because each escape would provide only a minor selective advantage [i.e., less than ρ(0)/*n*] (38, 39, 74).

Why is the virus not rejected by the strong immune reactions in our models? It is typically argued that HIV is not rejected because it escapes from immune responses and because it forms latently infected cells. Both mechanisms are absent from our equations. During the acute phase of the infection, the sizes of the immune responses are only limited by the availability of viral antigen, and the immune responses are expanding exponentially (albeit at a rate lower than that of the virus [47, 75]). The immune responses continue to expand (albeit even more slowly) after the peak in the viral load and when the viral load is rebounding after an initial phase of decline (Fig. 1 and 6). Since the total killing rate is monotonically increasing during the first weeks of the infection, the reason for the rebound in the viral load is that target cell levels recover following the peak in the viral load, allowing the virus to increase its effective replication rate.

Generally, CTL express high levels of the proteins associated with cytolytic activity (76). CD8^+^ T cells from the blood of HIV-infected patients also express high levels of perforin and form conjugates with autologous CD4^+^ T cells in *ex vivo* experiments, which in most cases leads to apoptosis of the CD4^+^ T cells (77). The *in vitro* cytotoxic capacity of CD8^+^ T cells on autologous HIV-1-infected CD4^+^ T cells is one of the best correlates with low HIV-1 loads in humans (78–80). Our modeling study confirms these experimental findings by showing that, if the immune pressure exerted by CD8^+^ T cells is largely cytolytic, the killing rate has to be much faster than is currently appreciated and that CTL should indeed play a major role in the death of infected cells. Another prediction of our modeling is that most of the killing should then either be early or late. This would be very important to know, but it is unclear how one could test this experimentally *in vivo*. Using *in vitro* cultures, it has been established that CTL that are specific for epitopes that are expressed early control HIV-1 replication better than CTL specific for a late protein (81). This suggests that early killing is beneficial (here because the CTL have more time to kill). Nowadays, time-lapse video imaging of target cells and CTL *in vitro* seems to be the most authoritative approach to establish when in the viral life cycle most infected cells are killed. Such experiments would need markers to know the status of the infected cells, as well as several clonotypes of CTL responding to epitopes that are expressed either early or late.

Like current experimental approaches, our modeling study fails to provide direct evidence that CD8^+^ T cells control HIV-1 infection by the killing of infected CD4^+^ T cells. We have only shown here that all experiments that were previously taken to favor nonlytic control by CD8^+^ T cells are perfectly consistent with a pure cytotoxic mechanism of control. Likewise, the excellent cytotoxic capacity of CD8^+^ T cells in patients with a very low viral load could be the consequence rather than the cause of low load (78–80). Therefore, we think it remains a crucial open question whether or not the immune control by CTL is largely lytic or nonlytic (and/or that CTL accrue nonlytic affects following killing a target [82]) and that one should not be convinced by the current circumstantial evidence, and we conclude with this important new insight that the current evidence is not conclusive.

## Figures and Tables

**FIG A1:**
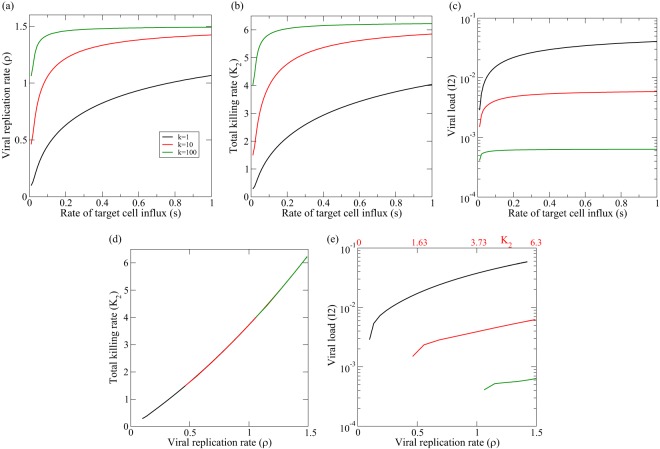
The realized killing rate *K*_2_ and replication rate ρ as a function of the *s* and *k_i_* parameters in the late-killing model. Panels d and e combine the graphs from panels a to c by plotting *K*_2_ or the set point viral load, *Ī*_2_, as a function of ρ. The fact that all lines fall on top of each other in panel d numerically confirms equation 18b. We use the function *F*(*T*) = *s* − *d_T_T* to complete d*T*/d*t* in equation 7a and the competitive monotonic saturation function *G*(*E_i_*,*V*) = *pVE_i_*/(*h_i_* + *V* + *E_i_*) with *V* = *I*_2_ to complete d*E_i_*/d*t* in equation 7b.

## APPENDIX

### Estimating the increase of the viral slope after CD8^+^ T cell depletion.

The viral replication rate, ρ, at steady state can be estimated from various sets of data on the increase in the viral load in chronically SIV-infected monkeys that are treated with antibodies depleting CD8^+^ T cells (9–15, 83, 84). Depending on the number of available time points, the slope, ρ, is estimated by linear regression of the log-transformed data or just from the ratios of pairs of log-transformed data points divided by their time interval. One complication in estimating ρ is that the antibody treatments were given over multiple days, and we start on the first day of depletion, taking it as the most significant depletion event. For the sake of clarity, the day corresponding to the first CD8^+^ T cell depletion treatment is here consistently referred to as day zero. The data from the Klatt et al. (11) and Chowdhury et al. (15) papers were kindly shared with us by the authors. The other data sets were included by digitizing the figures in the respective papers (using PlotDigitizer).

#### (i) The Schmitz et al. (9) data.

Already in 1999, CD8 depletion experiments were performed on rhesus macaques chronically infected with SIVmac (9). We digitized the data for the rhesus macaques denoted as A, B, and C. Measurements from days 0, 3, and 6 were available (Table A1). The average replication rate is ρ = 0.57 day^−1^.

**TABLE A1:** Slopes of the viral load in three chronically SIV-infected rhesus macaques in the data of Schmitz et al. (9)

Animal	Slope of viral load for days:		Linear regression
	0–3	3–6	
A	0.31	1.49	0.90
B	0.74	0.18	0.46
C	0.67	0.02	0.35
Mean	0.57	0.56	0.57

#### (ii) The Jin et al. (10) data.

In 1999 as well, similar experiments were performed on five rhesus macaques infected with SIVmac251 (animals AT-02, AR-68, AR-71, and AR-93) or SIVmac239 (animal AT-22) (10). Viral load data were collected on days 0, 1, and 2, and the corresponding slopes are presented in Table A2. The mean value of the slopes of the linear regressions is ρ = 0.58 day^−1^.

**TABLE A2:** Slopes estimated from the data of Jin et al. (10)

Animal	Slope for days:		Linear regression
	0–1	1–2	
AT-22	0.29	0.51	0.40
AR-68	0.48	1.12	0.80
AR-71	0.010	0.57	0.33
AR-93	0.037	0.40	0.39
AT-02	1.51	0.41	0.96
Mean	0.55	0.60	0.58

#### (iii) The Klatt et al. (11) data.

Two groups of SIVmac239-infected rhesus macaques were denoted as group A (animals RRf6, RAj7, RLi6, RPp6, and RZl5) and group B (animals RSq8, RUe7, RWf7, and XHB). Because there were no measurements of the viral load on day zero, we used its latest measurement prior to the CD8 depletion, which was day −2 in group A and day −6 in group B. Upslope data were available for day 1 and day 4 in group A and for day 1 and day 5 in group B. The estimates for the upslopes for the individual macaques and their means can be found in Table A3. The slopes obtained with linear regression seem the most reliable and have an average of ρ = 0.45 day^−1^.

**TABLE A3:** Estimates of the viral replication rate, ρ, from the data of Klatt et al. (11)

Group and animal	Upslope value of ρ for days:		Linear regression
	0–1	1–4	
A			
RRf6	1.59	0.73	0.85
RAj7	0.63	0.108	0.18
RLi6	2.05	0.31	0.56
RPp6	−0.14	0.16	0.12
RZl5	2.27	−0.13	0.21
Mean of group A	1.28	0.23	0.39
B	0–1	1–5	
RSq8	2.55	0.11	0.46
RUe7	1.58	0.57	0.72
RWf7	1.97	−0.27	0.047
XHB	2.03	0.76	0.95
Mean of group B	2.04	0.29	0.54
Mean of both groups	1.62	0.26	0.45

#### (iv) The Wong et al. (12) data.

Similar experiments were performed on eight rhesus macaques infected with SIVmac251 (12). We digitized the data for the three monkeys (MMU32906, MMU27562, and MMU33580) that approached full depletion of their CD8^+^ T cells prior to ART. Measurements were available for days 0 and 5, and we take the ratios of the log-transformed values, divide by 5, and obtain a very similar average of ρ = 0.54 day^−1^ (Table A4).

**TABLE A4:** Viral loads and slopes in three CD8-depleted rhesus macaques, from the data of Wong et al. (12)

Animal	ln*V* for day:		Slope
	0	5	
MMU32906	15.20	17.04	0.37
MMU27562	13.01	14.92	0.38
MMU33580	8.80	13.13	0.87
Mean			0.54

#### (v) The Pandrea et al. (13) data.

After a normal and vigorous acute infection, rhesus macaques infected with SIVagm control the infection and approach undetectable viral loads. Depleting the CD8^+^ T cells leads to rapid rebound of the virus. The upslope can only be estimated for two monkeys. Monkey BA38 had 3 consecutive time points above the level of detection and before the peak, and we estimate ρ = 0.7 day^−1^ from these data. Monkey V492 had two time points above the level of detection in the expansion phase, and we estimate ρ = 1.7 day^−1^ from these data.

#### (vi) The Fukazawa et al. (14) data.

As part of a larger study on B cell follicle sanctuaries, measurements were taken of the SIV RNA count in the plasma of seven elite-controller rhesus macaques after treatment with anti-CD8 antibodies (14). These monkeys were chronically infected with SIVmac239 or SIVmac251. The depletion treatment was not followed by ART, and remarkably, the monkeys were able to regenerate their SIV-specific CTL population and keep the virus in check. This is most likely due to the fact these monkeys were elite controllers, which is a vital difference with the four previous data sets. Data were taken on days 0, 3, 7, and 10 after the depletion treatments, and viral loads went down after day 10. We could only obtain data on the average values for all monkeys (days 0 to 3, ρ = 0.99; days 3 to 7, ρ = 0.94; and days 7 to 10, ρ = 0.56). As the last interval (days 7 to 10) showed a smaller increase than the first two intervals, linear regression was performed with and without this last interval, yielding slopes of ρ = 0.85 day^−1^ for 0 to 10 and ρ = 0.96 day^−1^ for days 0 to 7.

#### (vii) The Chowdhury et al. (15) data.

Different upslopes were observed following CD8 depletion in progressor and controller monkeys (15), with a significantly greater increase of the plasma viral load in controllers than in progressors. The means of the slopes for all intervals and the linear regression are higher in controllers than in progressor rhesus macaques (Tables A5 and A6). Linear regressions were obtained for days 0 to 2 and days 0 to 3 because the mean value of the slope between day 2 and day 3 is lower than for the other two intervals.

**TABLE A5:** Estimates for ρ from the controller monkeys in the study of Chowdhury et al. (15)

Controller animal	ρ for days:			Regression value for days:	
	0–1	1–2	2–3	0–3	0–2
RAk9	2.60	1.85	−1.11	1.18	2.22
RJm7	0.78	−0.80	1.16	0.26	−0.08
RLf8	4.39	0.97	−0.31	1.61	2.68
RSk8	2.84	2.62	1.50	2.35	2.73
RZc9	1.43	1.47	1.02	1.32	1.45
Mean	2.41	1.22	0.45	1.35	1.82

**TABLE A6:** Estimates for ρ from the progressor monkeys in the study of Chowdhury et al. (15)

Progressor animal	ρ for days:			Regression value for days:	
	0–1	1–2	2–3	0–3	0–2
RWm8	−0.14	NA^*a*^	0.01	−0.07	NA
RKv7	1.34	1.52	−0.35	0.91	1.43
RNw10	0.92	0.46	−0.07	0.44	0.69
RAy8	1.80	0.29	1.46	1.09	1.04
RLw10	1.32	0.37	−3.47	−0.49	0.85
RFn7	0.67	0.72	−0.39	0.37	0.70
RHd10	−0.07	0.33	0.11	0.14	0.13
RIc9	1.76	0.91	0.61	1.08	1.34
RDa10	1.92	1.43	0.19	1.21	1.68
RBc9	1.83	0.57	0.24	0.85	1.20
Mean	1.13	0.73	−0.18	0.55	1.00

a NA, not available.

To investigate whether this difference in upslopes between progressor and controller monkeys is significant, we performed a nonparametric Mann-Whitney U test. On the linear regressions based upon days 0 to 2, the test proved to be nonsignificant (*P* = 0.10). However, monkey RJm7 seemed to corrupt the data, since its viral load hardly increased upon CD8 depletion. If taken out, the results of the test indicated a very significant difference (*P* = 0.005). The same test was done for the slopes of the linear regression on interval days 0 to 3, and both with and without monkey RJm7, the test indicated a significant difference (*P* = 0.044 with RJm7 and *P* = 0.004 without RJm7).

#### (viii) The Metzner et al. (83) data.

Rhesus monkeys infected with SIVmac239Δnef for over 2 years underwent CTL depletion by treatment with OKT8F (anti-CD8) antibodies (83). We performed linear regression over the time points in which the CTL levels were undetectable, i.e., from day 0 to day 9 (but note that taking day 7, 8, or 10 as an endpoint hardly affects the estimates). Most of these monkeys have a very rapid increase in viral load during the first day after treatment, followed by a small decrease and a subsequent slower increase. The reasons for these fluctuations are unknown to us, and averaging by linear regression, we arrived at an estimate of ρ = 0.41 day^−1^. See Table A7 for all the values and means of the linear regressions performed.

**TABLE A7:** Estimates of the viral replication rate, ρ, from the data of Metzner et al. (83)

Monkey	Linear regression of ρ for days:			
	0–7	0–8	0–9	0–10
A	0.35	0.41	0.44	0.43
B	0.31	0.37	0.42	0.41
C	0.55	0.56	0.48	0.46
D	0.41	0.32	0.32	0.30
Mean	0.41	0.42	0.41	0.40

#### (ix) The Friedrich et al. (84) data.

A study was specifically conducted on six elite controller rhesus monkeys that had been chronically infected with SIVmac239 for between 1 and 5 years (84). The monkeys were depleted of their CTL, and a rapid increase in viral load in the blood plasma was observed. By performing linear regressions on the amounts of viral particles in the blood between treatment and 7 days after for the different monkeys, we arrived at an average regression of ρ = 0.96 day^−1^. One point in the data (for monkey AJ11 on day 3) could not be precisely determined from the figure, and hence, this monkey was not taken into account while estimating the value of the viral replication rate.

#### (x) Summary.

The average replication rates estimated from the studies of Schmitz et al. (9), Jin et al. (10), Klatt et al. (11), and Wong et al. (12) and from the progressors in the Chowdhury et al. (15) study vary in a narrow window between 0.45 ≤ ρ ≤ 0.6 day^−1^. Our conservative estimates for the elite controllers in the Fukazawa et al. (14), the Friedrich et al. (84), and the Chowdhury et al. (15) studies are about twofold larger and vary between 0.85 ≤ ρ ≤ 1.35 day^−1^. The two estimates from rhesus monkeys controlling SIVagm (13) to undetectable set point levels also tend to be high, i.e., ρ = 0.7 and ρ = 1.7.

### Target cell availability, viral load and replication, and killing rate.

If the two-stage model is extended with explicit functions for the production of target cells and immune effector cells, its steady state predicts correlations between viral replication, killing rates (equation 17), target cell availability, and viral load. In Fig. A1, we vary the production rate of target cells [*s*, while constraining *T*(0) = 1 by keeping *s* = *d_T_*] and the late killing rate k_2_1__ (setting *K*_1_ = 0) to study these correlations. (Similar results were obtained with the early killing regime [not shown]). The effective replication rate, ρ, increases with the killing rate and the renewal rate of the target cells (Fig. A1b), because a better immune control and a faster production of target cells increases the availability of target cells, *T̄*, which allows for a faster viral replication. The total killing rate, *K*_2_, also increases with the killing rate and the renewal rate of the target cells (Fig. A1b). The first is obvious, and the latter is natural, because a faster production of target cells needs to be compensated by a higher rate of killing at the steady state. The set point viral load increases with the rate of target cell production, *s*, and decreases with the killing rate k_2_1__ (Fig. A1c). Obviously, when the observed values of *K*_2_ and ρ are plotted as a function of each other (Fig. A1d), the six lines in Fig. 1a to c collide, as is predicted by equation 18b. Interestingly, markedly different viral loads can share identical values of ρ and *K*_2_ (Fig. A1e): for one *x* value, i.e., one combination of ρ and *K*_2_, we obtain 10-fold different viral loads with 10-fold changes in the killing parameter k_2_1__.

In biological terms, this can be understood by starting the argument either with the virus or with the immune response. First, patients infected with a virus that replicates fast, i.e., high ρ (due to viral properties and/or higher target cell availability by high immune activation), will approach a high set point viral load and, hence, elicit a large immune response that will ultimately balance the rapid viral replication. Second, patients mounting a good and broad immune response will control the virus well, which allows the target cells to recover, which in turn allows for rapid viral replication. Thus, rapid replication and killing are expected in controllers with a low viral load (15, 78–80) and in progressors with a high viral load (Fig. A1e). The fact that CTL appear to be less cytolytic in the latter group (78–80) could be indicative of exhaustion of CTL at high viral loads (85), which we could implement using a nonmonotonic proliferation function, *G*(*E_i_*,*V*); e.g., see Conway and Perelson (86). However, exhaustion of CTL would not change our general conclusion that all current evidence is consistent with rapid killing, and for reasons of clarity, we have used a monotonic proliferation function for the immune effector cells.
